# In situ eNOS/NO up-regulation—a simple and effective therapeutic strategy for diabetic skin ulcer

**DOI:** 10.1038/srep30326

**Published:** 2016-07-25

**Authors:** Ye Yang, Dengke Yin, Fei Wang, Ziyan Hou, Zhaohui Fang

**Affiliations:** 1 School of Pharmacy, Anhui University of Chinese Medicine, Hefei 230012, China; 2 Key Laboratory of Xin’an Medicine, Ministry of Education, Hefei 230031, China; 3 Department of Endocrinology, The First Affiliated Hospital of Anhui University of Chinese Medicine, Hefei 230031, China

## Abstract

Decreased nitric oxide (NO) synthesis and increased NO consumption in diabetes induces the inadequate blood flow to tissues that is primarily responsible for the pathogenesis and refractoriness of diabetic skin ulcers. The present study proposed a simple and effective therapeutic strategy for diabetic skin ulcers—*in situ* up-regulation of endothelial nitric oxide synthase (eNOS) expression and NO synthesis by statin-loaded tissue engineering scaffold (TES). *In vitro* experiments on human umbilical vein endothelial cells indicated that the statin-loaded TES relieved the high-glucose induced decrease in cell viability and promoted NO synthesis under high-glucose conditions. In a rat model of diabetes, the statin-loaded TES promoted eNOS expression and NO synthesis in/around the regenerated tissues. Subsequently, accelerated vascularization and elevated blood supply were observed, followed by rapid wound healing. These findings suggest that the *in situ* up-regulation of eNOS/NO by a statin-loaded TES may be a useful therapeutic method for intractable diabetic skin wounds.

Nitric oxide (NO) plays a key role in the physiological regulation of vascular functions, including vasorelaxation, anti-apoptosis, relief of oxidative stress, anti-inflammatory functions, stimulation of angiogenesis, and regulation of blood flow^1^,^2^. High serum concentrations of glucose and advanced glycation end products in people with diabetes induce vascular endothelial dysfunction (VED)^3^,^4^. VED results in reduced activation of endothelial nitric oxide synthase (eNOS) and increased reactive oxygen species, which account for the reduced synthesis and bioavailability of NO and increased NO consumption, respectively^5^,^6^. Various diabetes-related complications are associated with the consequently impaired vascular tone and inadequate delivery of oxygen and nutrients to tissues—including refractory skin ulcers^7^,^8^.

Many forms of specialized wound therapies have been applied to diabetic skin ulcers, such as flap transfer, bioengineered skin substitutes, recombinant growth factors, stem cells, and combinations thereof 9,10,11,12. Vascularization is a common factor restricting skin wound healing in diabetes. Given the crucial effects of NO on the pathogenesis and refractoriness of diabetic skin ulcers^7^,^8^ and the critical role of the eNOS/NO pathway in the action mechanism of pro-angiogenic factors^13^,^14^,^15^, the up-regulation of eNOS/NO should be a direct and effective strategy for accelerating skin ulcer healing in diabetes.

Statins are inhibitors of 3-hydroxy-3-methyl-glutaryl CoA reductase and have been widely used in patients with hypercholesterolemia. Furthermore, their pleiotropic effect of vasculature protection, in a manner independent of their lipid-lowering effect, has been demonstrated to be beneficial in reducing the risk of diabetic microangiopathy^16^,^17^,^18^. This protective effect of statins on endothelial function involves increasing NO biosynthesis and bioavailability via the direct up-regulation of eNOS expression^18^,^19^. Statins have also been demonstrated to increase the activity of endothelial progenitor cells and to promote the neovascularization of ischaemic tissue in normocholesterolemic animals^20^. Based on the studies above, a hypothesis regarding the applicability of statins in diabetic foot ulceration treatment and prevention has been proposed^21^. An early study has attempted to restore ischaemic limb blood flow in diabetic microangiopathy by intraperitoneal injection of pravastatin and pitavastatin^22^. However, daily injection is discomfort, whether through the enterocoelia or veins.

In recent decades, techniques of tissue engineering have attracted a great deal of attention in tissue/organ regeneration, tissue engineering scaffold (TES) fabrication is one of the most important component of which^23^. The mimicking of the extracellular matrix topology of TES is beneficial for cells migration and nutrients transport^24^. Additionally, the integration of drug delivery capacity into TES can provide sustained and effective bioactive substance to impaired tissues and promote the tissue regeneration^24^. In the current study, we attempted to implement *in situ* eNOS/NO up-regulation in/around skin wounds in a rat model of diabetes by using statin-loaded TESs. The *in vitro* protective effects of the statin-loaded TESs on the vascular endothelial cells were evaluated under high-glucose conditions and/or calcium ionophore stimulation, with cell viability and NO synthesis serving as indices. In the rat model of diabetes, statin-loaded TESs were administered on skin wounds. In addition to routine testings of glucose and low-density lipoprotein (LDL) levels in the blood, eNOS expression, NO synthesis and skin blood flow (SBF) in/around skin wounds, and the healing rate were evaluated. Diabetic skin wounds treated with blank TES, blank TES soaked with free statin, and natural healing were used as controls.

## Results

### Characterization of TES

**Figure 1:**
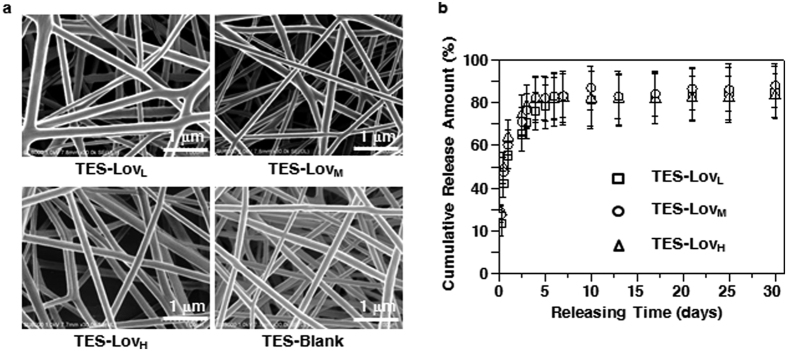
Morphology and *in vitro* drug release profile of TESs. (**a**) Representative SEM images of TESs with theoretical Lov loading amounts of 1.0% (TES-Lov_L_), 5.0% (TES-Lov_M_) and 10% (TES-Lov_H_) and blank TES without drug loading (TES-Blank). (**b**) *In vitro* release profiles for TES-Lov_L_, TES-Lov_M_ and TES-Lov_H_. All values represent the means and SD; *n* = 6.

Figure 1a shows the microscopic morphology of the TESs. Blank TES (group TES-Blank) and lovastatin (Lov)-loaded TESs with low (group TES-Lov_L_), medium (group TES-Lov_M_), and high drug loading (TES-Lov_H_) were all composed of uniform and randomly arrayed fibres, with diameters of 470 ± 105, 527 ± 80, 466 ± 117 and 550 ± 118 nm, respectively. The theoretical Lov loadings in TES-Lov_L_, TES-Lov_M_ and TES-Lov_H_ were 1.0%, 5.0% and 10.0%, respectively, where the actual loading amounts were 0.904 ± 0.002%, 4.13 ± 0.10% and 7.39 ± 0.40%, respectively. The encapsulation efficiencies of Lov in TES-Lov_L_, TES-Lov_M_ and TES-Lov_H_ were 89.83 ± 0.24%, 82.60 ± 1.93% and 84.33 ± 1.03%, respectively. The fibre diameter and encapsulation efficiency for each TES sample exhibited no significant differences (*p* > 0.05).

### *In vitro* drug release profiles from TESs

Figure 1b shows the cumulative release profiles of Lov from TES-Lov_L_, TES-Lov_M_ and TES-Lov_H_, all of which can be described as having two phases: a fast release phase in the initial 4 days and a very slow release in the later period. The cumulative amounts released during the initial 4 days from TES-Lov_L_, TES-Lov_M_ and TES-Lov_H_ were 76.0 ± 8.9%, 81.3 ± 10.5% and 82.2 ± 10.2%, respectively. The release profiles for the TES samples exhibited no significant differences (*p* > 0.05).

### *In vitro* effects of Lov-loaded TESs on high glucose induced cell damage in human umbilical vein endothelial cells (HUVECs)

**Figure 2:**
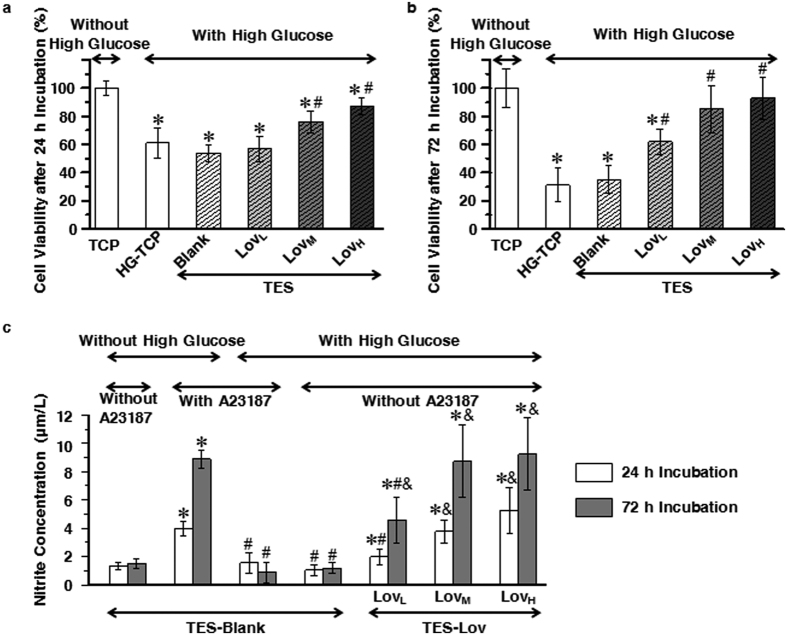
Effects of Lov-loaded TES on high-glucose induced cell damage in HUVECs. (**a,b)** Cell viability of HUVECs seeded on TCP, TES-Blank, TES-Lov_L_, TES-Lov_M_, and TES-Lov_H_ in high-glucose conditions, after 24 h (**a**) and 72 h (**b**) of incubation, with the cells on TCP in normal medium serving as controls. All values represent the means and SD; *n* = 6; *p < 0.05, *vs* TCP without high glucose; ^#^*p* < 0.05, *vs* TES-Blank with high glucose. (**c**) NO synthesis of HUVECs seeded on TES-Blank, TES-Lov_L_, TES-Lov_M_, and TES-Lov_H_ in medium with or without high glucose and A23187 after 24 and 72 h of incubation. All values represent the means and SD; *n* = 6; *p < 0.05, *vs* TES-Blank without high glucose or A23187; ^#^*p* < 0.05, *vs* TES-Blank with A23187 but without high glucose; ^&^*p* < 0.05, *vs* TES-Blank with high glucose but without A23187.

Figure 2a,b summarize the viability of HUVECs seeded on various materials in high-glucose conditions after 24 and 72 h of incubation. All cell viability data were converted into percentages, and data for cells on tissue culture plate (TCP) in normal conditions (without high glucose, group TCP) were set as at 100%. Cell viability on TCP (group HG-TCP) and TES-Blank in high-glucose conditions were significantly lower than that of cells on TCP in normal conditions (*p* < 0.05). As time passed, this high-glucose induced reduction became severe. However, there were no significant differences between these two groups (*p* > 0.05). In high-glucose conditions, the cell viability of group TES-Lov_L_ was equivalent to that of groups HG-TCP and TES-Blank after the initial 24 h of incubation (*p* > 0.05), and the viability was significantly higher after 72 h of incubation (*p* < 0.05). The cell viabilities of groups TES-Lov_M_ and TES-Lov_H_ in high-glucose conditions were always much higher than that of the HG-TCP group throughout the experiment (*p* < 0.05). Moreover, they were equivalent to that of group TCP in normal conditions after 72 h of incubation (*p* > 0.05).

Figure 2c summarizes the NO synthesis capabilities of HUVECs cultured in different conditions. The NO amount in the culture medium was quantified by detecting its metabolism of nitrite, owing to its instability. The results showed that the NO synthesis capability of HUVECs on TES-Blank was significantly enhanced by the calcium ionophore of A23187 (*p* < 0.05). However, this enhancement was completely eliminated under high-glucose conditions. Cells on the Lov-loaded TESs exhibited a much higher capability for NO synthesis than those on TES-Blank (*p* < 0.05) in high-glucose conditions. Furthermore, the TES-Lov_M_ and TES-Lov_H_ groups in high-glucose conditions exhibited NO synthesis equivalent to that of the TES-Blank group in normal medium with A23187 stimulation (*p* > 0.05).

### Establishment of the rat model of type I diabetes

**Figure 3:**
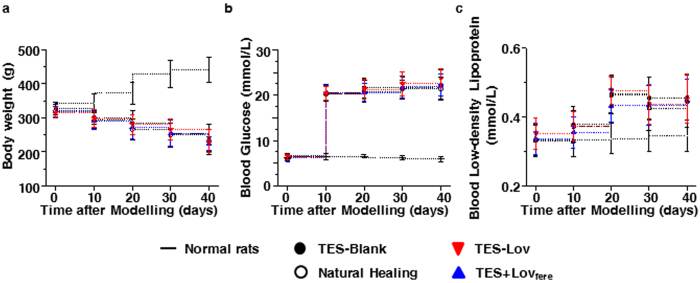
Physiological indices of rats after modelling. Changes in body weight (**a**), blood glucose (**b**), and blood LDL (**c**) in rats after modelling, with administration of TES-Blank, TES-Lov, or TES + Lov_free_ or natural healing, with the normal rats serving as controls. All values represent the means and SD; *n* = 6.

Because nearly 30% of the cases of streptozocin (STZ)-induced diabetes were self-healing in the pre-experiment, a large sample size was used in each group. The rats with blood glucose lower than 16.7 mM were removed. However, the sample size of each experimental group was always more than 6. Figure 3 summarizes the changes in body weight (Fig. 3a), blood glucose (Fig. 3b) and blood LDL (Fig. 3c) in the rats in each experimental group after modelling. Normal rats exhibited continued growth in body weight and retained stable blood glucose and LDL levels within the normal range. The diabetic rats in each experimental group displayed steadily decreased body weight and persistent hyperglycaemia and hyperlipidaemia after modelling. There were no deaths during the experiment.

### *In vivo* effects of Lov-loaded TESs on eNOS/NO during skin wound healing

**Figure 4:**
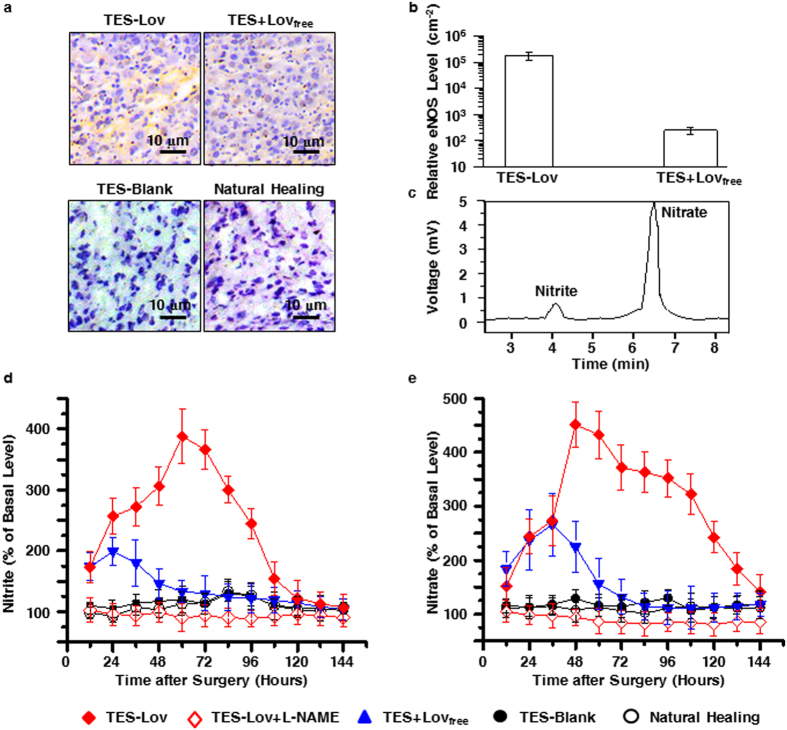
Effects of Lov-loaded TESs on eNOS expression and NO synthesis in regenerated tissues during skin wound healing. (**a**) Representative images of eNOS expression in regenerated skin tissues on the 3rd day after the administration of TES-Lov, TES + Lov_free_, TES-Blank, and natural healing. (**b**) Graphical representation of the relative eNOS level in groups TES-Lov and TES + Lov_free_ on the 3rd day after administration. (**c**) Chromatograms of nitrite and nitrate in the extract from regenerated skin tissue after 3 days administration of TES-Lov. (**d,e)** Temporal changes in extracellular nitrite (**d**) and nitrate (**e**) levels in the regenerated skin tissues after administration of TES-Lov, TES-Lov + L-NAME, TES + Lov_free_, TES-Blank, and natural healing. The resulting values were converted into percentages, with the value for samples from diabetic rats without skin wounds set at 100%. All image magnifications: ×400; scale bars: 10 μm. All values represent the means and SD; *n* = 6.

Given the *in vitro* effects of Lov-loaded TESs on NO synthesis in HUVECs, the TES-Lov_M_ was administered to skin wounds in diabetic rats. In order to discuss the advantages of drug entrapment into TES on tissue regeneration, Blank TES soaked with Lov solution were also covered on the skin wounds (group TES + Lov_free_). Figure 4a shows the immunohistochemistry (IHC) analysis of eNOS expression in regenerated skin tissues in each experimental group on the 3rd day after surgery. The TES-Lov group exhibited strong and extensive pale brown staining of eNOS, whereas the TES + Lov_free_ group exhibited sporadic staining, and the TES-Blank and NH groups did not exhibit staining. The relative level of positive eNOS staining in TES-Lov group was approximately 700 times higher than that of group TES + Lov_free_ (as shown in Fig. 4b).

Figure 4c shows the chromatogram of nitrite and nitrate extracted from the regenerated skin tissues. The extracellular levels of nitrite and nitrate around the skin wounds during the healing processes are shown in Fig. 4d,e. The NH and TES-Blank groups exhibited equivalent levels of nitrite and nitrate in intact skin during the healing processes. In the TES + Lov_free_ group, both the nitrite and the nitrate levels reached peak levels on the 1st day after surgery and decreased rapidly in the subsequent 2 days. The peak values of nitrite and nitrate in group TES + Lov_free_ were approximately 1.7 and 2.4 times those of the NH and TES-Blank groups. In the TES-Lov group, both the nitrite and the nitrate levels in/around the skin wounds increased rapidly from the beginning of the healing processes and reached the peak levels 60 and 48 h after surgery, which were approximately 3.7 and 4.2 times the durations of the NH and TES-Blank groups. Furthermore, the TES-Lov covered wounds in diabetic rats with eNOS inhibitor of N^ω^-nitro-L-arginine methyl ester (L-NAME) (group TES-Lov + L-NAME) exhibited extremely low levels of nitrite and nitrate in/around the skin wound, even lower than those of groups NH and TES-Blank.

### Effect of Lov-loaded TES on angiogenesis during skin wound healing

**Figure 5:**
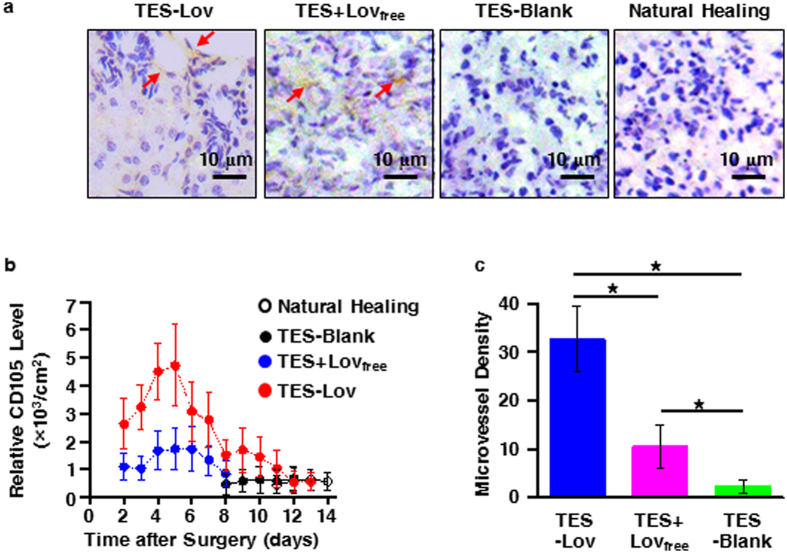
Effects of Lov-loaded TES on angiogenesis in regenerated tissues during wound healing. (**a**) Representative images of CD105 expression in regenerated skin tissues on the 5th day after administration of TES-Lov, TES + Lov_free_, TES-Blank, and natural healing. (**b**) Graphical representation of the relative CD105 level in the groups of TES-Lov, TES + Lov_free_, TES-Blank, and NH during the healing processes. (**c**) Graphical representation of MVD in regenerated skin tissues in groups TES-Lov, TES + Lov_free_, and TES-Blank. All image magnifications: ×400; scale bars: 10 μm. All values represent the means and SD; *n* = 6; *p < 0.05.

Figure 5a shows the IHC staining of endoglin (CD105) expression in regenerated skin tissues in each experimental group on the 5th day after surgery. The TES-Lov and TES + Lov_free_ groups exhibited pale brown staining of CD105, whereas the TES-Blank and NH groups did not exhibit staining. In addition, new vessels composed of flat epithelial cells were observed in sections of the TES-Lov group. Figure 5b summarizes the change curves of the relative CD105 level in the regenerated tissues of each experimental group during the initial 2 weeks of wound healing. The NH and TES-Blank groups exhibited extremely low levels of CD105 expression one week after surgery. Both the TES-Lov and TES + Lov_free_ groups exhibited rapid increases in CD105 expression from the beginning of wound healing and reached peak levels 5 days after surgery. However, the peak relative value of the positive stained CD105 in the TES-Lov group was approximately 3 times that in the TES + Lov_free_ group. Figure 5c shows the graphical representation of the microvessel density (MVD) of each experimental group on the 5th days after administration. The TES-Lov group exhibited nearly 3 times the MVD of the TES + Lov_free_ group.

### Assessment of SBF around the wound

**Figure 6:**
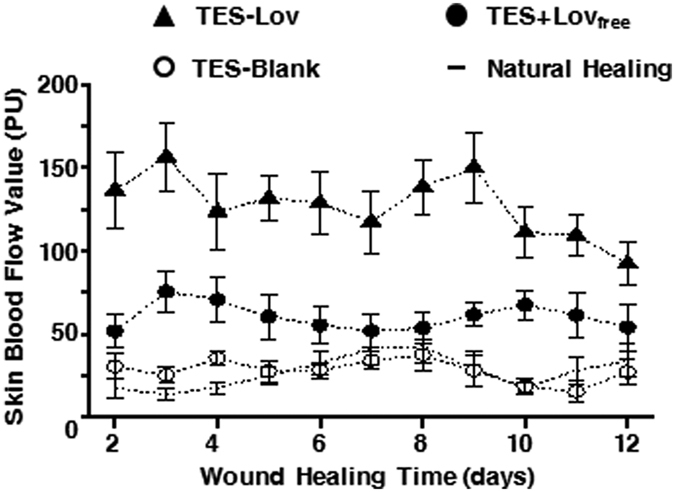
Change curves of SBF values around the skin wound during the healing process. Change curves of SBF values around the skin wound during the healing process with administrations of TES-Lov, TES + Lov_free_, TES-Blank, and natural healing. All values represent the means and SD; *n* = 6.

Figure 6 shows the change curves of the SBF value around the skin wounds during the healing process. In the NH and TES-Blank groups, the SBF values maintained a low level of approximately 25 PU. The SBF values of TES + Lov_free_ group were constant at approximately 50 PU during the healing process and exhibited an inconspicuous peak on the 3rd day after surgery. The TES-Lov exhibited much higher SBF values than the other groups (*p* < 0.05), with mean values nearly 3 and 6 times those of the TES + Lov_free_ and NH groups.

### Macroscopic observation of skin wound healing

**Figure 7:**
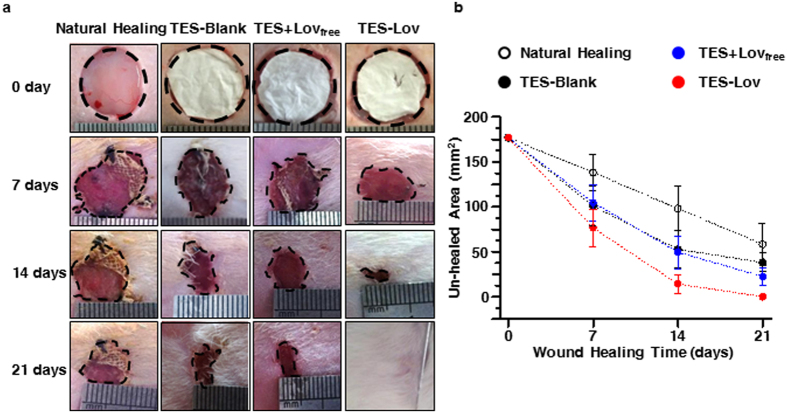
Effects of Lov-loaded TES on skin wound healing in the rat model of diabetes. (**a**) Representative images of the skin wounds on days 0, 7, 14, and 21 after administration of TES-Lov, TES + Lov_free_, TES-Blank, and natural healing. (**b**) Graphical representation of the un-healed areas of skin wounds in the TES-Lov, TES + Lov_free_, TES-Blank, and NH groups. All values represent the means and SD; *n* = 6.

Figure 7a,b shows the representative images and un-healed areas of skin wounds in each experimental group at different time points of the healing process. Skin wounds with TES-Lov exhibited much faster healing than those in the other groups (*p* < 0.05). After 7 days of administration, the TES-Lov group exhibited a reduction of the wound area. Nearly 56% of the wound area has been recovered, with new epithelia and sparse hair. The un-healed area was filled with granulation and no infection. Meanwhile, the NH, TES-Blank, and TES + Lov_free_ groups exhibited 22.6 ± 11.3%, 42.9 ± 12.4%, and 41.2 ± 11.6% wound closure, respectively, and the un-healed areas were dry and poorly granulated. After 14 days of administration, nearly 90% of the wounds in group TES-Lov were healed. The TES-Blank and TES + Lov_free_ groups exhibited similar healing rates of approximately 70%, which was much faster than that of the NH group. After 21 days of administration, the wounds in group TES-Lov were completely healed, and the regenerated skin was slick and ductile, similar to normal skin. In comparison, 33.0 ± 13.1%, 21.6 ± 5.8%, and 12.7 ± 5.2% un-healed areas were observed in the NH, TES-Blank, and TES + Lov_free_ groups, respectively.

## Discussion

This study demonstrates a simple and effective therapeutic strategy for diabetic skin ulcers, namely, the *in situ* up-regulation of eNOS/NO. This study is originated from the endothelial dysfunction that occurs in the development of refractory diabetic skin ulcers. Although the underlying mechanisms of the endothelial dysfunction in diabetes are complex and largely unknown, this dysfunction consistently occurs in both type-1 and type-2 diabetic patients^7^,^8^. Maintaining adequate perfusion to tissues is the primary function of the vascular system, and it is controlled by a balance between vasodilatation and vasoconstriction^7^. NO is the major vasodilatory factor for cutaneous vasodilatation^25^. Moreover, wound angiogenesis, the critical process of tissue formation and repair, is mediated by the synthesis of NO in endothelial cells^26^. In diabetes, reduced NO synthesis and bioavailability and increased NO consumption result in inadequate perfusion and decreased oxygenation and nourishment of tissues. Statins are drugs that are widely used for hyperlipidaemia. They also show positive effects on the progression of microvascular complications of diabetes by directly enhancing the ability of eNOS to generate NO thereby promoting the neovascularization of ischaemic tissue, in-dependently of their cholesterol-lowering actions^20^,^27^,^28^. Hence, statins should be effective for the treatment of diabetic skin ulcers by increasing blood perfusion to the tissues. Considering the mechanism and characteristics of diabetic skin ulcers, we applied a combination of statins and a TES to an animal model of diabetic skin wounds in the present study.

In the pre-experiments, high-glucose conditions exhibited dose-dependent cytotoxicity to HUVECs. Cell viability was the index showing the most apparent changes. Although the data for the pre-experiments are not presented in this article, the results corresponded to those of previous studies^29^. The similar data for cells on TCP and TES-Blank in high-glucose conditions, which were much lower than those for cells on TCP in normal conditions, indicated that decreased cell viability resulted from high-glucose conditions rather than the polymeric TES. The recovery of cell viability in TES-Lov_M_ and TES-Lov_H_ groups, especially after 72 h of incubation in high-glucose medium, showed that Lov has a protective effect against high-glucose induced cell damage in HUVECs. Analysis of the NO synthesis potencies of HUVECs, A23187 showed a time-dependent improvement of NO production in normal conditions, as shown in Fig. 2c. However, this NO improvement by A23187 was significantly suppressed by high-glucose levels. The much higher NO synthesis in cells seeded on Lov-loaded TES than those seeded on Blank-TES indicated that Lov promoted NO synthesis in a manner unaffected by high-glucose conditions.

Because TES-Lov_M_ and TES-Lov_H_ exhibit similar protection against high-glucose induced cytotoxicity in HUVECs, the TES with medium Lov loading was administered to skin wounds in diabetic rats. The same levels of body weight, blood glucose and LDL in all the diabetic groups during wound healing (as shown in Fig. 3) indicated that the restoration of microvascular function in the present study was unrelated with to the hypolipidaemic effect of Lov.

In contrast with the persistent and significant up-regulation of Lov-loaded TES on eNOS expression, NO production, and SBF values in/around the skin wounds and the marked acceleration of the healing rate, only temporary and low-degree promotions were observed in the TES + Lov_free_ group. Moreover, the healing rate of the TES + Lov_free_ group was similar to that of the TES-Blank group. These results indicated that the addition of free Lov in a single administration could not work effectively because of its rapid loss. Besides these, the prevention of NO synthesis in group TES-Lov + L-NAME indicated that the therapeutic effect of TES-Lov on skin wound in diabetic rat model might occur at least partly via the eNOS/NO pathway.

In diabetic vascular complications, systemic administration of statins is common. In previous studies, statins have been used to restore ischaemic limb blood flow in diabetic mice through intraperitoneal injection^22^. Although significant prevention of autoamputation has been achieved, the complexity and discomfort of injection cannot be neglected. In addition, clinical data in recent years suggest a possible association between statins use and incident diabetes in patients with underlying diabetes risk factors^30^. However, statins have not been clearly shown to increase diabetic microvasular complication^31^. Furthermore, the topically delivery of statins from TES could not only provide a sustained release of statins to the target location but also avoid the systemic harmful effects.

Many active substances have been combined with TESs in attempts to restore the VED of diabetic skin ulcers, such as proteins^32^, polypeptides^33^, genes^34^, polysaccharides^35^, and cells^36^. Most of them have achieved the expected requirement of promoting angiogenesis and accelerating wound closure. However, the remaining activity is typically the major restricting factor for the fabrication and storage of these active substances. Statins, whether fungus-extracted, semisynthetic, or synthetic, exhibit a stable chemical structure and activity during regular preparation and storage processes. This work focuses on the restoration of inadequate blood flow and angiogenesis during skin wound healing in diabetes. eNOS/NO, the action target of statins, is the key factor in the signalling pathways of angiogenesis^37^. Other wound healing mechanisms are also triggered by NO, which may be modulated by sustained release of statins from the TES, involving inflammation and cell proliferation^38^.

Lov is a representative lipophilic statin with confirmed protective effects on endothelial cells^39^. Previous research has suggested that the administration of lipophilic statins lead to much higher up-regulation of eNOS expression than does the administration of hydrophilic statins^22^. This is probably due to the easy diffusion of lipophilic statins to tissues. Because the drug-loaded TES can provide direct and efficient drug delivery to target tissues, we speculate that the hydrophilic statins may also be effective for the treatment of skin wounds in diabetes.

## Materials and Methods

### Materials

Poly(lactide-co-glycolide) (PLGA, lactide:glycolide = 65:35, *M*_w_ = 62 kDa) was obtained from Puluo Jiayuan Biomedical Material Limited Company (Zhejiang, China). Lov, STZ, L-NAME, heparin, bovine serum albumin (BSA) and HEPES buffer were from Sigma-Aldrich (St. Louis, MO). Rabbit anti-mouse antibodies specific to CD105 and eNOS, biotinylated secondary antibody, streptavidin-horseradish peroxidase (HRP) and 3,3′-diaminobenzidine (DAB) were purchased from Biosynthesis Biotechnology Co., Ltd (Beijing, China). All other chemicals were of analytical grade and were purchased from Zhongshi Chemical Engineering Company (Shanghai, China).

### Ethics statement

The animal experimental protocol was reviewed and approved by the Institutional Ethics Committee of the Anhui University of Chinese Medicine. Experiments were carried out in accordance with the approved guidelines.

### Preparation of statin-loaded TESs

Lov and PLGA were dissolved in chloroform by vigorous stirring with the following ratios of Lov to PLGA to chloroform: 0.7: 7: 100, 0.35: 7: 100, and 0.07: 7: 100 (w/w/v). The homogeneous Lov-PLGA/chloroform solution was loaded into a 2 mL glass syringe and electrospun by an electrospinning setup comprising a high-voltage statitron (DC 30000V, Tianjin Dongwen High Voltage Power Supply Co., Ltd, Tianjin, China) and a precision pump (LSP01-1A, Baoding Longer Precision Pump Co., Ltd, Hebei, China). The electrospinning parameters of voltage, exit orifice diameter, nozzle velocity, and receiving distance were 15 kV, 0.6 mm, 1.0 mL/h, and 15 cm, respectively. The resulting Lov-loaded TESs were designated as TES-Lov_H_, TES-Lov_M_, and TES-Lov_L_ respectively. A control sample without Lov was also prepared using the above parameters above and named TES-Blank. All obtained TESs were vacuum-dried at room temperature for 2 days to remove any solvent residue.

### Characterization of TESs

TES-Lov_H_, TES-Lov_M_, TES-Lov_L_ and TES-Blank were mounted on metal stubs, sputter-coated with gold, and then observed with a Representative scanning electron microscope (SEM, FEI Quanta 200, the Netherlands). The diameters of the compositional fibers of each TES were calculated by using Adobe Photoshop CS program (Adobe Systems, Inc., CA, USA) from five SEM images.

The loading amount and encapsulation efficiency of Lov into the TESs were determined by dissolving the TES in chloroform and detecting the Lov content using high-performance liquid chromatography (HPLC). In brief, a known amount of Lov-loaded TESs (ca. 10 mg) was dissolved in chloroform (500 μL), centrifuged at 15,000 × g for 15 min and filtered with a 0.22 μm microfiltration membrane. Lov content was detected using an HPLC system consisting of a Shimadzu Essential LC-15C Separations Module, an SPD-15C Dual Absorbance Ultraviolet Detector, an SIL-10AF Autosampler, a CTO-15C Column Oven, and an Inertsil ODS (5 μm × 4.6 mm × 250 mm) column (SHIMADZU, Japan) with a mixed mobile phase of acetonitrile and 0.1% phosphoric acid (60: 40, v/v) at flow rate of 1.0 mL/min, column temperature of 40 °C, and detected wavelength of 238 nm. The concentration was obtained using a standard curve prepared from known concentrations of Lov solution. The Lov loading efficiency was obtained through comparing the amount in electrospun TES with the initial adding amount.

### *In vitro* stains release profiles from TESs

Thirty milligrams of Lov-loaded TESs were immersed in 10 mL of phosphate buffer (PBS, 10 mM, pH 7.4), containing 0.5% (w/w) of sodium dodecyl sulfate (SDS), and kept in a thermostat water bath (Taichang Medical Apparatus Co., Jiangsu, China), that was maintained at 37 °C and 100 cycles/min. At predetermined time intervals, 1.0 mL of release buffer was removed for analysis, and 1.0 mL of fresh PBS (with 0.5% SDS) was added to continue the incubation. The Lov amount present in the release buffer was detected using the HPLC method described above. The amount of Lov in the release buffer taken out for detection was also added into the cumulative release total. TES-Blank was used as a control for the evaluation of Lov release.

### Effect on high glucose-induced cell damage in HUVECs

HUVECs (ATCC No. CRL-1730, passages 3-6) were maintained in Ham’s F12K medium (Gibco-BRL, Paisley, UK) containing 0.1 mg/mL heparin, 0.03 mg/mL endothelial cell growth supplement (Gibco BRL, Grand Island, NY), and 10% foetal bovine serum (Gibco BRL, Grand Island, NY) at 37 °C in a CO_2_ incubator (Thermo Forma 370, Thermo Fisher Scientific, Inc., Waltham, MA, USA).The medium was changed every 2-3 days. After reaching 80–90% confluence, the HUVECs were trypsinized, passaged, and seeded on a 12-well TCP or TES.

Cell seeding on the TESs was performed in accordance with previous research^40^. In brief, the TESs were cut into small disks (25 mm in diameter and 5 mg in weight), fixed by a cell-culture ring designed by Zhu *et al*.^41^, sterilized by electron-beam irradiation using a linear accelerator (Precise, Elekta, Crawley, U.K.) with a total dose of 100 cGy, soaked in the cell culture medium, and placed in a 12-well TCP. A total of 400 μL of the HUVEC suspension (1 × 10^5^ cells/mL) was seeded onto the presoaked TESs. The cell-seeded TESs were incubated at 37 °C, 5% CO_2_, and 95% relative humidity for 4 h to make the cells diffuse into and adhere to the TES. Then, 3.5 mL of culture medium was added into each well.

In a pre-experiment, HUVECs were treated with medium containing various concentrations of glucose. The results showed that a glucose level higher than 25 mM could significantly reduce the cell viability and NO synthesis of the HUVECs. Thus, a glucose concentration of 30 mM was chosen to induce cell damage in subsequent experiments. To evaluate the effects of TESs on HUVEC viability under high-glucose conditions, equal numbers of cells were seeded on TES-Lov_H_, TES-Lov_M_, TES-Lov_L_, and TES-Blank. After 4 h of cell adhesion, 3.5 mL medium containing 30 mM glucose was added into each well. The viability of HUVECs on the TESs was assayed with a cell counting kit (CCK-8, Dojindo Molecular Technologies, Inc., Kumamoto, Japan) after 24 and 72 h of incubation. Briefly, all TESs were rinsed, moved to another TCP, and immersed in 400 μL of fresh normal culture medium. Then, 40 μL of CCK-8 reagent was added into each well. After 2 h of incubation, 150 μL of incubated medium was pipetted into a 96-well TCP, and the absorbance at 450 nm was measured using a μQuant microplate spectrophotometer (Elx-800, Bio-Tek Instrument, Inc., Winooski, VT). Cells seeded on the TCP and incubated in normal or high-glucose medium were set as the normal control (group TCP) and the high-glucose control (group HG-TCP), respectively. All experiments were performed with *n* = 6.

To evaluate the effects of the TESs on NO synthesis in HUVECs under high-glucose conditions, A23187, a receptor-independent eNOS agonist, was used as a stimulant of NO production^42^,^43^,^44^. In a pre-experiment, the HUVECs were treated with medium with various concentrations of A23187. The results showed that A23187 significantly increased NO synthesis in HUVECs in a concentration-dependent manner. Thus, a concentration of 2.5 μM was used to induce NO synthesis in HUVECs in the subsequent experiments. In brief, equal numbers of cells were seeded on TES-Lov_H_, TES-Lov_M_, TES-Lov_L_, and TES-Blank. After 4 h of cell adhesion, the TES-Blank was randomly divided into four groups (*n* = 6 in each group) and immersed in 3.5 mL of medium with or without 30 mM glucose and 2.5 μM A23187. Meanwhile, 3.5 mL of medium with high glucose but without A23187 was added into the wells (*n* = 6 in each group). Cell medium was harvested 24 and 72 h later and assayed with an NO detection kit (Beyotime Institute of Biotechnology, Nanjing, China). Briefly, 50 μL of incubated medium was pipetted into a 96-well TCP, and 50 μL of Griess Reagents I and II were subsequently added. Absorbance at 540 nm was measured using a μQuant microplate spectrophotometer. The NO concentration in the medium was calculated using a standard curve from a known concentration of sodium nitrite solution.

### Creation of skin wounds on diabetic rats and treatments

The rat model of type I diabetes was established as described previously^45^. Briefly, Sprague-Dawley rats (male, 250–300 g, 10 weeks of age, purchased from the Medical University of Anhui) were intraperitoneally injected with 45 mg/kg STZ (dissolved in sodium citrate buffer, pH 4.5). Blood glucose levels were monitored using a complete blood glucose monitor (Shenzhen Radiant Innovation Co., Ltd., Shenzhen, China), and rats were considered to be diabetic when levels were higher than 16.7 mM^45^. To ensure a hyperglycaemic throughout the experiment, blood glucose levels were monitored every ten days after modelling. Fifty microliters of blood was collected from the eye socket vein every ten days after modelling and LDL in the blood was detected using an automated biochemical analyser (7600, Hitachi, Japan). Body weight was measured at the same time.

The dorsal area of each diabetic rat was totally depilated by 8.0% (w/v) Na_2_S and scissored with four full-thickness circular wounds (*d* = 1.2 cm) after the rat was anesthetized with 45 mg/mL pentobarbital. The skin wounds were covered with TES-Blank (10.0 mg), TES-Lov (10.0 mg), TES + Lov_free_ (10.0 mg TES-Blank soaked with 200 μL PBS containing 400 μg free Lov), with natural wound healing (NH group) as the control. Sterilized gauze was applied to each wound and sutured to the skin around the wound. To inhibit NO synthesis, 6 more diabetic rats were removed the dorsal skin and administrated with TES-Lov as mentioned above and orally administered with L-NAME (1 mg/mL in drinking water)^22^.

### IHC staining

To evaluate angiogenesis in the regenerated skin tissues during the healing process, eNOS and CD105 expression was detected by IHC. The regenerated skin tissues were collected and frozen sectioned every day during the initial 2 weeks. The sections were gradually immersed in H_2_O_2_ (3%, v/v) and citrate buffer solution (10 mM, pH 6.0), heated in a microwave oven, washed with PBS (10 mM, pH 7.2), and blocked with BSA in Tris-buffered saline (5%, w/v). The excess liquid was drained, and the sections were incubated with primary antibodies at 4 °C overnight, washed with PBS (10 mM, pH 7.2), and sequentially incubated with biotinylated secondary antibody for 20 min, streptavidin-HRP for 20 min, and DAB-H_2_O_2._ The sections were then counterstained with haematoxylin, dehydrated, hyalinised and mounted. The IHC-stained sections were observed with a light microscope (BX41-12P02, OLYMPUS, Japan), and the relative eNOS/CD105 level of pale brown colour were calculated with Image Tool program (V. 3.0, The University of Texas Health Science Center, San Antonio, TV). In briefly, five images of each IHC-stained section were randomly picked under the same magnification. The pale brown colour of positive eNOS/CD105 staining was marked by the software automatically. The pixel values per square centimeter were calculated, averaged and defined as relative eNOS/CD105 level. The mean value of the six samples in each experimental group was calculated and a relative eNOS/CD105 level-time curve was prepared. For the MVD, an endotheliocyte cluster of pale brown colour of CD105 expression was identified as a blood vessel, regardless of whether the lumen or red blood cells were present, according to the method of Weidner^46^.

### *In vivo* microdialysis

Diabetic rats with skin wounds and various treatments were anesthetized using pentobarbital and maintained at 37 °C. Heparin sodium (100 U/mL) treated microdialysis probes (CMA 30 linear probe, CMA Microdialysis AB, Kista, Sweden) were connected via polyethylene tubing to a microsyringe driven by a syringe pump (CMA 4004, CMA Microdialysis AB, Kista, Sweden) at one end, and punctured into the dermis layer close to the wound edges at the other end. Phosphate buffer (10 mM, pH 7.4) was perfused at a flow rate of 2.0 μL/min. After 1 h perfusion to reach an equilibrium state, the perfusates were collected from the outlet of the probe via polyethylene tubing. Microdialysis was performed every 12 h after skin wound modelling. After 12 cycles of perfusate collection, each animal was decapitated, the skin tissues around the wound were removed, and the accuracy of the probe placements in the dermis layer was verified histologically. Because of the relative state of the technique of microdialysis, 6 more diabetic rats without skin wounds were set as control group and sampled as the method mentioned above.

### Measurements of nitrite and nitrate

Owing to its instability, the quantitative analysis of NO production in/around the skin wounds was performed by simultaneously measuring the NO metabolites of nitrite (NO_2_^−^) and nitrate (NO_3_^−^) in the microdialysate. The method for measuring NO_2_^−^ and NO_3_^−^ levels was described previously^47^. Briefly, 10 μL of the microdialysate fraction was injected into an ENO-30 NO-detector (Eicom, San Diego, CA). The NO_2_^−^ and NO_3_^−^ in the microdialysate were separated by a reverse-phase separation column (NO-PAK, 4.6 × 50 mm, Eicom, San Diego, CA), and NO_3_^−^ was reduced to NO_2_^−^ in a reduction column (NO-RED, Eicom, San Diego, CA) at 35 °C column temperature. Then, the NO_2_^−^ was reacted with Griess reagent, which was delivered at a rate of 0.1 mL/min, to form a purple azo dye in a reaction coil at 35 °C. The absorbance of the product dye was detected at 540 nm by a flow-through spectrophotometer (NOD-10, Eicom, San Diego, CA). The mobile phase was purchased from Eicom and delivered at a rate of 0.33 mL/min. The concentrations of NO_2_^−^ and NO_3_^−^ in the microdialysate were obtained using a standard curve prepared from known concentrations of sodium nitrite and sodium nitrate. The resulting values were converted into percentages, taking the values for samples from diabetic rats without skin wounds as 100%. All experiments were performed with *n* = 6.

### Assessment of SBF around the wound

The SBF values around the wound during the healing process were investigated with laser Doppler fluximetry (moorVMS-LDF, Moor Instruments Ltd., Axminster, United Kingdom). The diabetic rats were randomized into 2 groups (*n* = 6 in each group), and a circular full-thickness skin wound (*d* = 1.2 cm) was created on each side of the spine. The right wounds of the two groups were treated with TES-Lov and TES + Lov_free_, and the left wounds were treated with TES-Blank and natural healing. Beginning on the 2^nd^ day after the surgery, the SBF values around the skin wounds were assessed daily as previously described^48^,^49^. In brief, the rats were lightly anesthetized, acclimatized, gently cleaned and rested prostrate. One probe was attached approximately 5 cm from the wound edges for a baseline, avoiding hair or visible blood vessels, and the other probe was attached on the edge of the wound. The data were detected by a dual channel laser Doppler monitor (Moor Instruments Ltd., Axminster, United Kingdom) and recorded by MoorVMS-PC V2.0 software, with a resolution of 40 points/second and a normalization of the baseline flux.

### Macroscopic observation of skin wound healing

All the skin wounds were photographed every 7 days after the surgery. Wound areas without epidermis cover were defined as un-healed area and were manually marked. The un-healed areas were calculated by using Image Tool program.

### Statistical analysis

The results were expressed as means ± standard deviation of percentage values of the basal level (*n* = 6). Statistical significance of differences was examined using one-way analysis of variance (ANOVA) followed by LSD post hoc test. A *p* value of < 0.05 was considered to be statistically significant.

## Additional Information

**How to cite this article**: Yang, Y. *et al*. In situ eNOS/NO up-regulation—a simple and effective therapeutic strategy for diabetic skin ulcer. *Sci. Rep.* **6**, 30326; doi: 10.1038/srep30326 (2016).
